# Slower alpha rhythm associates with poorer seizure control in epilepsy

**DOI:** 10.1002/acn3.710

**Published:** 2018-12-18

**Authors:** Eugenio Abela, Adam D. Pawley, Chayanin Tangwiriyasakul, Siti N. Yaakub, Fahmida A. Chowdhury, Robert D. C. Elwes, Franz Brunnhuber, Mark P. Richardson

**Affiliations:** ^1^ Department of Basic and Clinical Neuroscience Institute of Psychiatry, Psychology and Neuroscience King's College London London United Kingdom; ^2^ Department of Clinical Neurophysiology King's College Hospital NHS Foundation Trust London United Kingdom; ^3^ Social, Genetic and Developmental Psychiatry Research Center Institute of Psychiatry, Psychology and Neuroscience King's College London London United Kingdom; ^4^ School of Biomedical Engineering & Imaging Sciences King's College London London United Kingdom; ^5^ National Hospital for Neurology and Neurosurgery UCL Hospitals NHS Foundation Trust London United Kingdom

## Abstract

**Objective:**

Slowing and frontal spread of the alpha rhythm have been reported in multiple epilepsy syndromes. We investigated whether these phenomena are associated with seizure control.

**Methods:**

We prospectively acquired resting‐state electroencephalogram (EEG) in 63 patients with focal and idiopathic generalized epilepsy (FE and IGE) and 39 age‐ and gender‐matched healthy subjects (HS). Patients were divided into good and poor (≥4 seizures/12 months) seizure control groups based on self‐reports and clinical records. We computed spectral power from 20‐sec EEG segments during eyes‐closed wakefulness, free of interictal abnormalities, and quantified power in high‐ and low‐alpha bands. Analysis of covariance and post hoc *t*‐tests were used to assess group differences in alpha‐power shift across all EEG channels. Permutation‐based statistics were used to assess the topography of this shift across the whole scalp.

**Results:**

Compared to HS, patients showed a statistically significant shift of spectral power from high‐ to low‐alpha frequencies (effect size *g* = 0.78 [95% confidence interval 0.43, 1.20]). This alpha‐power shift was driven by patients with poor seizure control in both FE and IGE (*g* = 1.14, [0.65, 1.74]), and occurred over midline frontal and bilateral occipital regions. IGE exhibited less alpha power shift compared to FE over bilateral frontal regions (*g* = −1.16 [−0.68, −1.74]). There was no interaction between syndrome and seizure control. Effects were independent of antiepileptic drug load, time of day, or subgroup definitions.

**Interpretation:**

Alpha slowing and anteriorization are a robust finding in patients with epilepsy and might represent a generic indicator of seizure liability.

## Introduction

The human alpha rhythm is a prominent oscillatory electroencephalographic signal seen over occipital recording sites during relaxed, eyes‐closed wakefulness. It is thought to arise through cortico‐thalamic interactions, and to possibly reflect top‐down processes that subtend a vast number of cognitive operations, in particular attention and working memory.^1^ The importance of the alpha rhythm is underscored by its alterations in neurological disease, where it typically slows down and loses its characteristic anterior‐to‐posterior gradient – changes that are usually commensurate with clinical severity and therefore useful to monitor neurological dysfunction.^2^

These alterations of alpha, which are often seen over longer stretches of the electroencephalogram (EEG), are considered to be the hallmark of diffuse cortical‐subcortical neuropathology, such as metabolic or neurodegenerative diseases, but they have been found in epilepsy as well. In fact, studies conducted during the first decades of EEG research found conspicuous alpha rhythm alterations in epilepsy patients; specifically, they reported that both power and topography of the alpha rhythm were shifted: power from higher (8–13 Hz) to lower (6–9 Hz) frequencies, and topography from occipital to frontal sites.^3^, ^4^ Subsequent quantitative EEG studies confirmed these alterations in a wide variety of focal epilepsy (FE) and idiopathic (or genetic) generalized epilepsy (IGE) syndromes, and have identified them in unaffected first‐degree IGE relatives and in drug‐naïve patients as well.^5^, ^6^, ^7^, ^8^, ^9^, ^10^ The cause of this phenomenon and its clinical relevance remain poorly understood.

Because alpha rhythm alterations have been reported in such a disparate variety of epilepsy syndromes, they might simply reflect an unspecific byproduct of disease. Alternatively, they could point to shared neurobiological substrates for seizure generation. Although this might seem counterintuitive, given the multitude of insults and genetic mutations that can lead to seizures, a wealth of neuroimaging studies now supports the idea that icto‐ and epileptogenic mechanisms ultimately converge on a set of large‐scale neural networks, in which thalamus and midline frontal and parietal cortices play an important role, even if they do not contain the seizure focus.^11^ Alpha alterations could therefore reflect pathological resting‐state dynamics of this core network, and its liability to seizures. In fact, our group has previously shown that EEG networks from IGE patients show increased functional connectivity in low‐alpha frequencies,^12^ which associate with an increased propensity to generate seizure‐like oscillations.^13^ Does alpha slowing therefore indicate that the brain is operating closer to seizure threshold?

To address this question, we quantitatively assessed the distribution of alpha power between high‐ and low‐frequency bands in resting‐state EEG recordings of healthy subjects (HS), and FE and IGE patients with good and poor seizure control (PSC). We hypothesized that seizure control, but not epilepsy type or antiepileptic drugs (AED) load, would determine the degree of alpha‐power shift and its topography, suggesting shared ictogenic mechanisms across epilepsy syndromes.

## Methods

### Participants

We analyzed data from 102 subjects investigated at King's College Hospital (KCH), London, between 2008 and 2015: 39 were healthy volunteers recruited through a local volunteer database, and 63 were patients with epilepsy (44 outpatients and 19 inpatients). Patients were recruited from KCH and collaborating hospitals, and included if they were over 18 years of age and had a definite diagnosis of epilepsy made by an experienced epileptologist on the basis of clinical presentation, EEG, and MRI. Diagnosis and classification were made in accordance with the International League Against Epilepsy (ILAE) 2017 classification of epilepsies and seizures.^14^, ^15^ All controls and 9 IGE patients have been included in previous studies.^12^, ^16^

We divided patients into subgroups based on clinical presentation (IGE vs. FE syndromes) and their level of seizure control, which we determined from seizure self‐reports. This information was corroborated with clinical notes and electronic patient records whenever possible. We defined good seizure control (GSC) as fewer than four seizures of any type during the 12 months prior to study inclusion, and PSC otherwise. We also verified that no patient had had a seizure 24 h prior to EEG recording.

This study was approved by the KCH research ethics committee (ref. 08/H0808/157) and the Bromley research ethics committee (ref. 12/LO/2030). All participants gave written informed consent before enrolment.

### Clinical data

We collected the following variables: gender, age, disease duration, syndrome type and lateralization, seizure types and frequency over 12 months, AED number and dosage, total AED load, and the presence of potentially epileptogenic MRI lesions. Total AED load was calculated by taking, for each drug, the ratio between its prescribed daily dose, and the defined daily dose (as determined by the WHO Collaborating Centre for Drug Statistics Methodology, http://www.whocc.no/atc_ddd_index/.) and then summing over all AED.^17^

### EEG recordings

Ten minutes of awake EEG data were recorded on a NicoletOne system (Viasys Health Care, San Diego, CA) at 256 Hz from 19 channels positioned according to the international 10–20 system, with two reference electrodes attached to the ear lobes. The same EEG technologist performed all HS and outpatient measurements, using the same recording room and system for all participants. Inpatient EEGs were performed by a variety of staff members of the epilepsy monitoring unit at KCH, according to clinical schedules. If participants consented, hyperventilation and photic stimulation were carried out.

### EEG visual analysis

Three EEG‐trained neurologists (F.C., R.E., and F.B.) reviewed all EEGs and noted the following phenomena: presence of focal interictal epileptiform discharges (IED), generalized spike‐wave discharges, focal slowing, and normal variants. F.C., R.E., and A.P. then selected two segments of 20 sec of eyes‐closed, awake EEG from each participant for subsequent analysis, which had to be free of large movement artefacts, epileptiform discharges, and signs of drowsiness or sleep. The first segment was used for the main analyses described below, the second was held out for control analyses. Data review and selection were done before data analysis. Further data analysis was carried out by E.A., who was not involved in selection of EEG segments.

### EEG power spectrum

EEG data were processed using Fieldtrip (Version 20171203, http://www.ru.nl/neuroimaging/fieldtrip)^18^ running on MATLAB (R2016b, The MathWorks, Inc., Natick, MA). EEG segments were first re‐referenced to a common average. We then calculated the power spectra of each segment using a Hanning‐tapered fast Fourier transform (FFT). To this end, each segment was cut into a series of 1 sec epochs with 90% overlap, demeaned, multiplied with the Hanning‐taper, and zero‐padded before computing the FFT. Power was calculated by squaring the modulus of the Fourier coefficients for each channel from 2 to 20 Hz in steps 1 Hz, and then normalized against total power for each channel. We chose this frequency range because it provided a broad overview of frequency bands commonly used in the literature, while avoiding very low‐frequency drifts and eye movements (<2 Hz), as well as muscle artefacts (>20 Hz).^19^ To test our hypotheses, we focused on the low‐alpha (6–9 Hz) and high‐alpha band (10–11 Hz).^20^ This choice was motivated by band‐definitions used in previous work from our group.^12^, ^13^

### Statistical analysis

Clinical data were analyzed using JASP (Version 0.8.6, https://jasp-stats.org/) or SPSS (Version 24, IBM, UK). We report continuous variables as mean ± SD (range) and binary variables as proportions. We used the Shapiro–Wilk test to assess deviations from normality. If assumptions were met, we compared means using unpaired *t*‐tests, else we applied the Mann–Whitney *U*‐test (or the Kruskal–Wallis test in the case of three groups). We compared proportions using the *χ* ^2^‐test or Fisher's exact test if the number of observations in any cell was ≤5.

The analysis of EEG power spectra had three aims: first, to confirm that there was a shift in the alpha‐power spectrum from higher to lower frequencies as previously reported, second, to test whether this effect was driven by patients with PSC, over and above syndrome classifications, and third, to assess whether the alpha‐power shift occurred not only over occipital electrodes, where the alpha rhythm usually predominates, but over frontal electrodes as well. To simplify calculations, we defined the “alpha‐power shift” as our outcome variable, that is the ratio of average power in the low‐alpha power (6–9 Hz) over average power in the high‐alpha (10–11 Hz):

$$\text{Alpha}-\text{power shift}=\frac{\mu_{\mathrm{Low}-\mathrm{alpha}\;\mathrm{power}}}{\mu_{\mathrm{High}-\mathrm{alpha}\;\mathrm{power}}}$$

This measure increases if power in low‐alpha frequencies increases, and will decrease otherwise. Collapsing over multiple frequencies and using a ratio has the advantage of reducing the number of multiple comparisons.

We performed all analyses both on grand‐average power spectra and on scalp topographies. For the grand‐average analyses, we averaged the power spectra across all channels and then calculated the power shift as above. This yielded one value per subject, that is a measure of global alpha‐power shift without topographical information. Statistical analysis was then carried out in JASP statistical software using these values as dependent variable. We first computed a one‐way between‐subjects ANCOVA to test for differences in alpha‐power shift between groups, with age and gender serving as covariates (which were included because of their known effects on EEG power spectra).^21^ Other clinical covariates were omitted because they were confounded with group assignment (i.e., only patients were treated with AEDs), and thus would have reduced model degrees of freedom without explaining clinically meaningful differences. We assessed the equality of variances using Levene's test, and the assumption of the homogeneity of regression slopes by modelling interaction terms between the group variable and each covariate. Furthermore, we inspected Q‐Q plots of residuals to assess their departure from normality. We then used an *F*‐test to determine the main effect of group, and post hoc *t*‐tests to assess the difference between the three subgroups. We next conducted a 2 × 2 between‐subjects factorial ANCOVA on the patient data only, with the first factor syndrome (FE or IGE) and the second factor seizure control (GSC or PSC), with age, gender and total AED load serving as covariates. AED load has been shown to increase low‐frequency power in previous patient studies^5^, ^22^; it was thus essential to include it as a covariate in here. Assumptions checks and statistical tests were conducted as above. We used a significance threshold of *P* < 0.05, family‐wise error (FWE) corrected for multiple comparisons (Bonferroni).

For the topographic analyses, we calculated the alpha‐power shift for each channel individually, and then linearly interpolated these values to produce, for each subject, a 2D scalp map on a regular 32 × 32 mm spatial grid.^23^ Maps were then smoothed with an 8 × 8 mm^2^ Gaussian kernel to account for the relatively sparse spatial sampling.^23^ Statistics on these maps were carried out with Permutation Analysis of Linear Models (PALM, version alpha109, https://fsl.fmrib.ox.ac.uk/fsl/fslwiki/PALM).^24^ We calculated pair‐wise comparisons between (1) HS and all patients, (2) GSC and PSC patients, and (3) IGE and FE patients, using unequal variances *t*‐test at each map element (or voxel), with age and gender serving as covariates for all models, and AED load as an additional covariate for between‐patient comparisons. This yielded one T‐map per comparison (we did not recalculate the *F*‐tests on spatial maps to avoid redundancy). To assess statistical significance without distributional assumptions, we ran permutations tests, that is recalculated T‐maps after shuffling subjects between groups, using the Freedman‐Lane algorithm to account for the presence of covariates (10 000 permutations).^24^ We set the significance threshold at *P* < 0.05, FWE‐corrected based on the empirical *t*‐value distribution derived from permutations.

To quantify effect sizes, we derived *η* ^*2*^ and Hedge's *g* for all *F*‐ and *t*‐tests, respectively. Hedge's *g* has the same interpretation as Cohen's *d*, but is less biased in the presence of unequal sample sizes.^25^ We used *η* ^*2*^ as provided from JASP output, and calculated Hedge's *g* using the Measure of Effect Size toolbox (https://github.com/hhentschke/measures-of-effect-size-toolbox).

### Control analyses

We conducted a number of control analyses to rule out confounds (see Data S1). These included (1) repeating the analyses above using individual alpha frequency (IAF) instead of alpha‐power shift, (2) assessing whether comparable effects could be reproduced in a second data segment of the same EEG recording, (3) testing two alterative seizure‐control stratifications, that is grouping patients according to whether they had no versus any seizures and according to a median split, (4) testing a direct correlation between seizure frequency and alpha‐power shift, without subgroup dichotomization, (5) investigating circadian influences by comparing EEG recording times between groups, (6) checking for potential effects of specific AEDs, if their load varied between groups, and (7) repeating the topographical analysis in non‐lesional epilepsy syndromes to rule out that spatial alterations were an epiphenomenon of structural damage.

### Classification analysis

Post hoc, we also explored whether alpha‐power shifts could be used to predict subject‐wise seizure control and used a 10‐fold cross‐validated linear discriminant analysis (LDA) to classify GSC and PSC patient data.^26^

### Data visualizations and resources

Plots of average spectra were generated with Gramm,^27^ high‐resolution scalp topographies with SPM12 and Fieldtrip. Code and processed data are publicly available on https://osf.io/f2vya/. Raw EEG data can be obtained from the senior author upon request.

## Results

### Clinical features

Table 1 summarizes clinical data. There were more patients with a focal syndrome in the PSC compared to the GSC group. In keeping with this finding, PSC patients suffered more often from focal impaired awareness seizures, presented more focal IED, and more often MRI lesions. On the other hand, GSC patients were treated on average with higher doses of Lamotrigine compared to PSC patients. Photic stimulation and hyperventilation were performed only in a subset of patients and were inconspicuous in all of them (photic: 15 GSC, 14 PSC; hyperventilation: 12 GSC, 13 PSC).

**Table 1:** Demographic and clinical characteristics in HS, epilepsy patients with GSC, and epilepsy patients with PSC

	HS (*n* = 39)	GSC (*n* = 25)	PSC (*n* = 38)	Statistic	*P*
Female sex, *n* (%)	19 (49)	15 (60)	19 (50)	*χ* ^2^(2, 102) = 0.87	0.647
Age, y	30 ± 9 (18–53)	33 ± 12 (20–77)	38 ± 14 (20–68)	H = 4.62	0.099
Disease characteristics					
Disease duration, y	‐	16 ± 10 (1–42)	17 ± 15 (2–58)	*U* = 424.5	0.482
FE syndrome, *n* (%)	‐	10 (40)	27 (71)	*χ* ^2^ (1, 63) = 6.00	0.014
FE left lateralized, *n* (%)	‐	5 (20)^a^	14 (37)^a^	Fisher's exact test	0.175
FE right lateralized, *n* (%)		3 (12)	12 (32)	Fisher's exact test	0.129
GTCS, *n* (%)	‐	4 (16)	12 (32)	Fisher's exact test	0.233
AS, *n* (%)	‐	4 (16)	4 (11)	Fisher's exact test	0.394
FIAS, *n* (%)	‐	1 (4)	24 (62)	Fisher's exact test	<0.001
FAS, *n* (%)	‐	0 (0)	2 (5)	Fisher's exact test	0.513
EEG characteristics					
Background slowing, *n* (%)	‐	1 (4)	3 (8)	Fisher's exact test	0.999
Focal slowing, *n* (%)	‐	4 (16)	15 (39)	Fisher's exact test	0.055
GSWD, *n* (%)	‐	5 (25)	10 (26)	Fisher's exact test	0.764
IED, *n* (%)	‐	4 (19)	15 (39)	Fisher's exact test	0.002
MRI characteristics^b^					
Lesion, *n* (%)	‐	3 (14)	17 (50)	Fisher's exact test	0.005
MTS, *n* (%)	‐	2 (8)	6 (16)	Fisher's exact test	0.246
Medication					
AED, *n*	‐	1 (1–3)^c^	2 (2–3)	*U* = 375.9	0.119
AED drug load, a.u.	‐	1.4 ± 0.9 (0.4–4.3)	1.5 ± 0.8 (0.2–4.0)	*U* = 417.0	0.418
Patients on LTG, *n* (%)	‐	10 (40)	12 (32)	Fisher's exact test	0.802
LTG dosage, mg	‐	350 ± 91 (200–450)	242 ± 120 (50–400)	*t*(20) = 2.3^d^	0.030
Patients on LEV, *n* (%)	‐	7 (28)	11 (29)	Fisher's exact test	0.999
LEV dosage, mg	‐	1500 ± 595 (750–2500)	1550 ± 934 (300–3000)	*t*(16) = −0.13	0.902
Patients on VPA, *n* (%)	‐	8 (32)	10 (26)	Fisher's exact test	0.789
VPA dosage, mg	‐	838 ± 307 (300–1200)	1000 ± 531 (300–2000)	*t*(16) = −0.77	0.455
Patients on CBZ, *n* (%)	‐	6 (24)	10 (26)	Fisher's exact test	0.999
CBZ dosage, mg	‐	833 ± 497 (400–1400)	690 ± 277 (200–1000)	*U* = 35.0	0.618
Others, *n*	‐	2 ETX, 1 LAC, 1 TGB, 1 TPM, 1 ZNS	2 LAC, 1 OXC, 3 PHT, 1 TGB, 6 TPM, 2 ZNS	‐	‐

Numbers are given as mean ± SD (range), unless stated otherwise, and all *P*‐values are two‐tailed. AED names: CBZ, carbamazepine; ETX, ethosuximide; LAC, lacosamide; LEV, levetiracetam; LTG, lamotrigine; OXC, oxcarbazepine; PHT, phenytoin; TGB, tiagabine; TPM, topiramate; VPA, valproic acid; ZNS, zonisamide. HS, healthy subjects; GSC, good seizure control; PSC, poor seizure control; *χ* ^2^, Chi‐square test (degrees of freedom, sample size); H, Kruskal–Wallis tests; *U*, Mann–Whitney‐*U*‐test; FE, focal epilepsy; GTCS, generalized tonic‐clonic seizures; AS, absence seizures; FIAS, focal impaired awareness seizures; FAS, focal aware seizures; EEG, electroencephalogram; GSWD, generalized spike‐wave discharges; IED, interictal epileptiform discharges; MRI, magnetic resonance imaging; MTS, mesial temporal lobe sclerosis; AED, antiepileptic drugs; a.u., arbitrary units; *n*, number; *t*‐test (degrees of freedom); y, years.

a Lateralization was unclear for two GSC and one PSC patient with FE.

b MRI reports were not available in one patient with good, and four patients with PSC.

c Median (range).

d *T*‐tests where used if data met normality assumptions, *U*‐tests otherwise.

### Alpha‐power shifts occur in patients with PSC in both focal and generalized epilepsy syndromes, and do not depend on age, gender, or drug load

**Figure 1:**
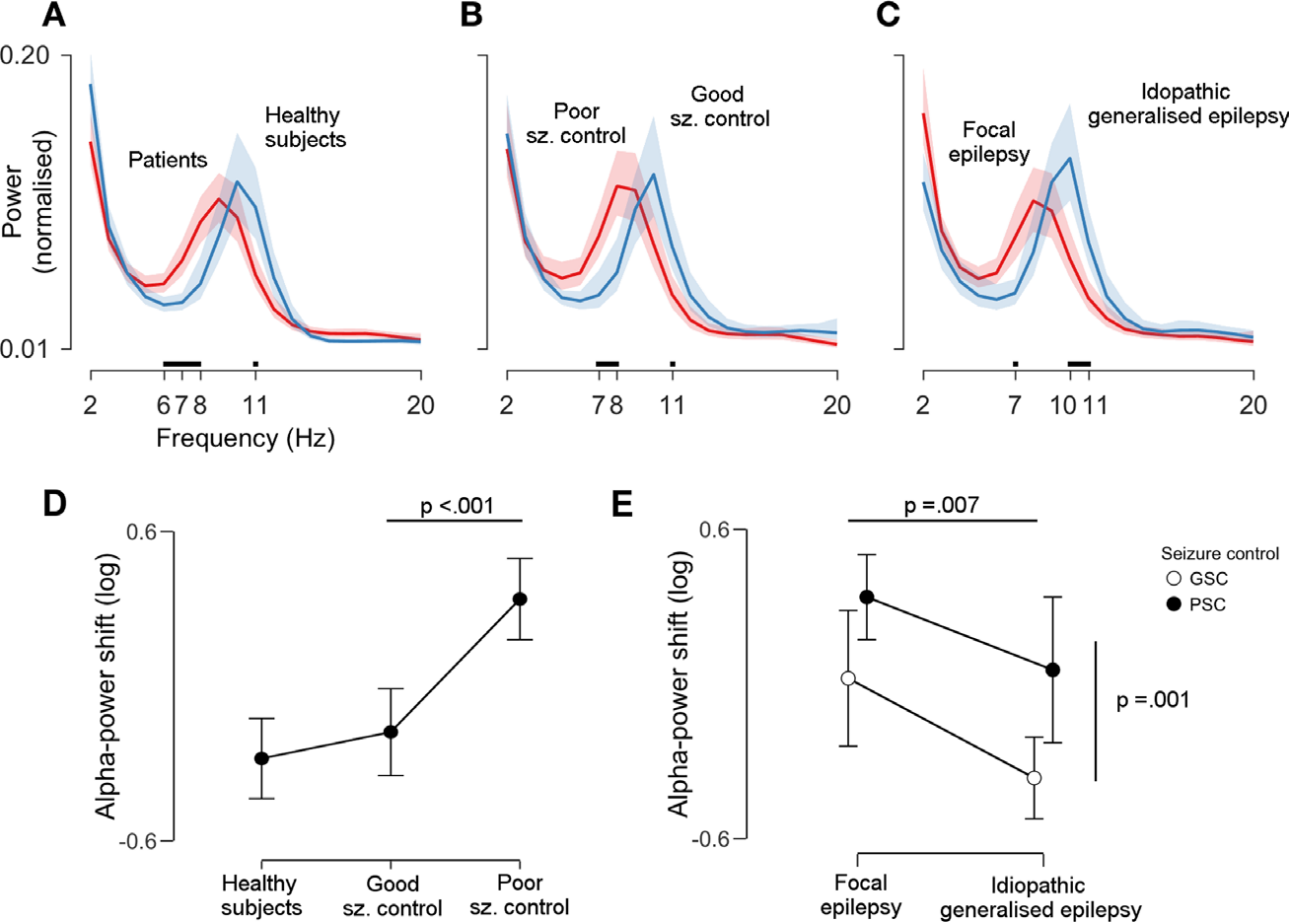
Alpha power is shifted towards lower frequencies in patients with poor seizure control across clinical syndromes. The three plots in the upper row (A–C) compare the following pairs of power spectra (from left to right): patients versus healthy subjects, patients with poor versus good seizure control (GSC), and patients with focal versus idiopathic generalized epilepsy. Lines indicate group averages and shaded areas 95% confidence intervals (CI). Tick marks and black lines above the *x*‐axis show frequencies at which power spectra diverge: a shift towards lower alpha frequencies can be appreciated for the whole patient cohort, for poor seizure control, and for focal epilepsy subgroups. Plots (D and E) show the statistical assessment of this observations in terms of the (log‐transformed) alpha‐power shift, that is the ratio of average low‐ to average high alpha‐power. Higher values indicate more low‐alpha power. Dots and error bars represent means ± 95% CI. *P*‐values are derived from pair‐wise contrasts of two analyses of covariance (see Methods for details). There was a significant difference between healthy subjects and patients, which was driven by poor seizure control patients (*P* < 0.001, panel D). The power spectrum of focal epilepsy patients was more shifted than the power spectrum of idiopathic generalized epilepsy patients (*P* = 0.007, panel E, horizontal line). However, there was no syndrome‐by‐seizure control interaction: alpha power in poor seizure control patients was always more shifted than alpha power in GSC patients (*P* = 0.001), and this occurred in equal measure in both syndrome categories (panel E, vertical line).

Visual inspection of average power spectra revealed a shift of EEG power from high‐ to low‐alpha in all subgroup comparisons (Fig. 1A–C). To quantify these observations, we first calculated a one‐way between‐subjects ANCOVA comparing HS against both PSC and GSC, with age and gender serving as covariates. Levene's test was significant (*P *<* *0.001); this was ameliorated after taking the binary log of the alpha‐power shift (*P *=* *0.268). Q‐Q‐plot of residuals showed no departure from normality, and tests for the assumption of homogeneity of regression slopes revealed no interaction between covariates and groups (gender × group, *P *=* *0.307, age × group, *P *=* *0.520), indicating that model assumptions were met. After partialling out variance associated with age and gender, there was a statistically significant difference in alpha‐power shift between groups, *F*(2, 97) = 17.20, *P *<* *0.001, *η* ^*2*^ = 0.26. Planned contrasts revealed that patients presented a clear alpha‐power shift compared to HS (M* *=* *−0.32, SD = 0.48), *t*(100) = 3.62, *P *<* *0.001, *g *=* *0.78, 95% CI [0.43, 1.20]. This effect was driven by PSC patients (M =* *0.28, SD = 0.48), which had a substantially larger shift than the GSC group (M* *=* *−0.24, SD = 0.41), *t*(61) = 4.23, *P *<* *0.001, *g *=* *1.14, 95% CI [0.65, 1.74] (Fig. 1D).

We next assessed whether alpha‐power shifts were a unique feature of PSC patients, regardless of the underlying syndrome. To do so, we conducted a 2 × 2 between‐subjects factorial ANCOVA with the first factor syndrome (FE or IGE) and the second factor seizure control (GSC or PSC), with age, gender and total AED load serving as covariates. Again, tests for homogeneity of variances and regression slopes were not significant (all *P* > 0.05). We found statistically significant main effects of syndrome, *F*(1, 59) = 7.74, *P *=* *0.007, *η* ^*2*^ = 0.099, and seizure control, *F*(1, 57) = 11.65, *P *=* *0.001, *η* ^*2*^ = 0.15, but no statistically significant interaction, *F*(1, 57) = 0.477, *P *=* *0.492, *η* ^*2*^ = 0.006. Planned contrasts indicated that FE patients (M* *=* *0.27, SD = 0.48) presented a larger alpha‐power shift than IGE patients (M* *=* *−0.20, SD = 0.45), *t*(61) = 2.78, *P *=* *0.007, *g *=* *1.16 [95% CI 0.67, 1.74]. Within each syndrome category, PSC patients showed significantly larger shifts than their GSC counterparts, *t*(61) = 3.41, *P *=* *0.001, *g *=* *1.12 [95% CI 0.64, 1.70] (Fig. 1E).

### Alpha‐power shifts are topographically extended, indicating a forward spread of low‐alpha power

We next asked whether there was a topographical shift, that is a forward spread of the low‐frequency alpha rhythm, as well. To do so, we calculated pairwise comparisons between groups using topographical maps of alpha‐power shift and assessed their significance with permutation tests.

**Figure 2:**
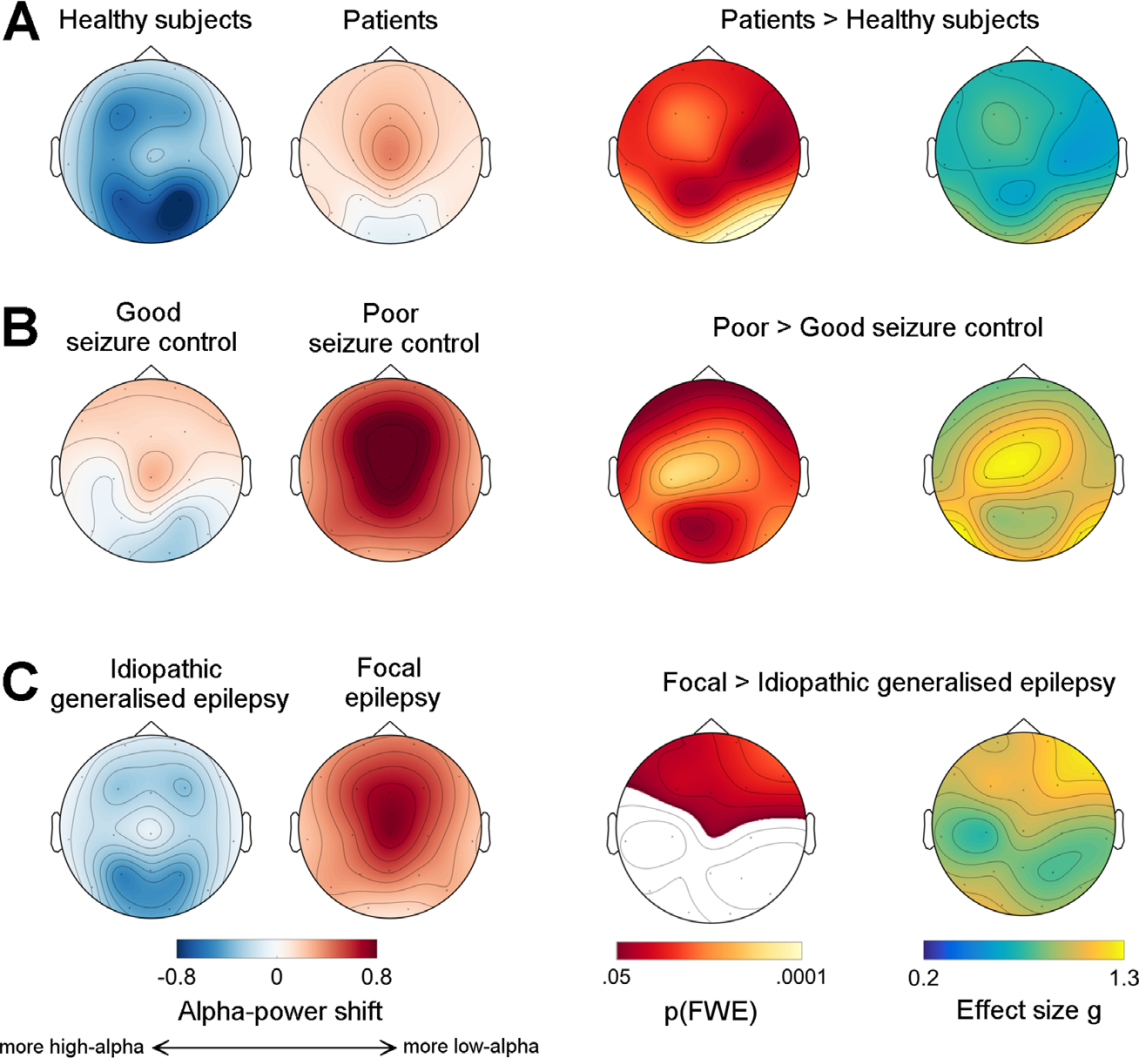
Alpha‐power shifts are topographically extended, indicating a forward spread of low‐alpha power. This figure shows topographical maps for three pair‐wise comparisons: panel (A), healthy subjects versus all epilepsy patients, panel (B), good‐seizure control versus poor‐seizure control patients, and panel (C), idiopathic generalized epilepsy versus focal epilepsy patients. The first two columns on the left show raw data: positive values (red) indicate a shift of electroencephalogram (EEG) power towards the low‐alpha band, negative (blue) values a shift towards the high‐alpha band. The third and fourth column show statistical maps, that is *P*‐values and effect sizes, respectively. Maps of family‐wise error (FWE) corrected *P*‐values were derived using permutation tests. White areas did not reach the significance threshold. The last column shows effect size maps (Hedges’ *g*), where *g* = 0.2 represents a weak, and *g* = 1.3 a very large effect. Note that all patients present significant shifts of alpha power compared to healthy controls over the entire scalp (panel A), with large effects occipitally, and that this effect is particularly pronounced in poor seizure control patients: very large effects are seen here over bilateral and midline frontal regions (panel B). Finally, *t* focal epilepsy patients had more frontal alpha‐power shift, that is more low‐alpha power, over frontal regions compared to idiopathic generalized epilepsy patients, (panel C).

This analysis revealed that alpha‐power was shifted in patients towards lower frequencies both frontally and occipitally, with a maximum over the right occipital region (Table 2, Fig. 2A). Again, this effect was driven by PSC patients, who presented an increased alpha‐power shift that broadly covered the entire scalp, with peaks over central and bilateral occipital regions (Table 2, Fig. 2B). The comparison between IGE and FE patients revealed that FE patients had more alpha‐power shift over both frontal regions, with a maximum on the right (Table 2, Fig. 2C).

**Table 2:** Statistical results for alpha‐power shift topographies

Factors	Covariates	Contrast	Peak statistic (df)	*P*(FWE)^a^	Effect size *g* [95% CI]^b^	Nearest electrode
Group (HS, GSC patients, PSC patients)	Age, gender	PAT > HS	*t*(97) = 4.52	<0.001	1.03 [0.65, 1.47]	T6
Seizure control (GSC, PSC)	Age, gender, AED load	PSC > GSC	*t*(56) = 4.31	<0.001	1.28 [0.86, 1.80]	C3
Syndrome (IGE, FE)	Age, gender, AED load	IGE < FE	*t*(56) = 2.72	0.031	−1.15 [−0.65, −1.81]	O1

Electrode names follow the standard 10–20 system. FWE, family‐wise error; df, degrees of freedom; CI, confidence interval; GSC, good seizure control; PSC, poor seizure control; PAT, all patients; HS, healthy subjects; AED, antiepileptic drugs; IGE, idiopathic generalized epilepsies; FE, focal epilepsy.

a *P*‐values have been FWE corrected using Gaussian random fields.

b Effect size measure based on mean differences divided by the pooled and weighted standard deviation. Interpretation: 0.2 = small, 0.5 = medium; 0.8 = large, 1.2 = very large effect. Confidence intervals were derived from 5000 bootstrap samples.

### Control analyses confirm a general slowing down of the alpha‐rhythm, and rule out data selection or acquisition biases as alternative explanations

We conducted a number of control analyses to test the robustness of our findings to methodological choices. Full results are summarized in Data S1. Briefly, comparing patients on the basis of IAF instead of power lead to qualitatively similar results, as did analyzing a second data segment from the same recording. Furthermore, PSC patients were always more shifted compared to GSC patients, even when using alternative dichotomization rules or accounting for patients on lamotrigine (which showed between‐group dosage differences). Of note, we also found a significant correlation between individual alpha‐power shift and seizure frequency, particularly over frontal recording sites, thus corroborating the subgroup analyses above. In addition, there were no significant differences in terms of EEG recording times, ruling out circadian confounds. Finally, both focal PSC patients without discernible brain lesions, as well as IGE‐PSC patients, which are non‐lesional per definition, showed anteriorization of low alpha‐power.

These analyses thus show that findings were fairly consistent over a number of approaches, and robust to confounds.

### Alpha‐power shift can discriminate seizure control subgroups

An exploratory LDA with 10‐fold cross‐validation achieved the following performance parameters (mean ± SD [95% CI]): sensitivity of 71.2 ± 3.9% [63.5%, 78.8%], specificity of 79.6 ± 2.2% [75.2%, 83.9%], and AUC of 78.8 ± 1.0% [67.7%, 89.8%], which was highly significant (*z*‐test, *P* = 1.7 × 10^−7^).

## Discussion

In this study, we show that the power spectrum of the alpha rhythm is shifted towards lower frequencies in epilepsy patients with PSC, in both focal and generalized epilepsy syndromes. Furthermore, we demonstrate that this effect extends from occipital to frontal regions, and is more pronounced in FE patients. Our study took major steps to control for methodological confounds. First, we explicitly included age, gender and AED load as covariates in all our models, and used permutation‐based statistics for assessing scalp EEG topographies, thus achieving adequate false‐positive control with few assumptions. Second, we confirmed that our results were robust to data selection procedures, patient subgroup definitions, circadian effects, and different outcome metrics (power vs. frequency). Finally, we could show that spatial alterations in alpha‐power occurred also in non‐lesional epilepsy syndromes. Taken together, the present analyses indicate that space‐frequency alterations of the alpha rhythm strongly associate with increased seizure liability in common epilepsy syndromes, whether generalized or focal.

The observation that alpha rhythm is altered in epilepsy is not new. In fact, a number of historic and contemporaneous studies have described slower alpha rhythms in heterogeneous (focal and generalized) epilepsy cohorts, sometimes pointing out the anteriorization of topography as well.^3^, ^4^, ^5^, ^6^, ^8^ Capitalizing on recent advances in permutation statistics and topographical EEG analysis,^23^, ^24^ our study advances this line of research by showing that this seemingly ubiquitous phenomenon is likely related to seizure liability: significant alpha‐power shifts were present in all patients when compared to healthy controls, but were specifically driven by patients with PSC. Effect sizes were moderate to very large, indicating that these findings were not subtle in quantitative terms. Importantly, analyses were done on data segments that were considered normal by experts, suggesting that ongoing background EEG rhythms contain clinically valuable information that cannot be gleaned from visual analysis alone. This information that could have practical utility: a post hoc, exploratory classification analysis suggests that patients with poor versus good seizure‐control might be identified at the individual level with fair accuracy (AUC ~0.8) on the basis of alpha‐power shifts alone. This performance is comparable to a recent report that used highly optimized classifiers on resting‐state EEG data to discriminate between epilepsy patients and neurological controls,^5^ although larger, multi‐center data sets are certainly needed to confirm this result.

Another important aspect is that alpha‐power shifts were present in equal measure both in FE and IGE syndromes, that is, there was no significant syndrome‐by‐seizure control interaction (parallel slopes in Fig. 1E). This means that, independent of the underlying clinical syndrome, patients with PSC exhibit more alpha‐slowing and frontal spread than well‐controlled patients, although power in FE patients was more shifted overall (Fig. 1E), and there were clear topographic differences of this effect between IGE and FE patients (Fig. 2C). These findings suggest that alpha‐rhythm alterations might reflect seizure‐promoting mechanisms that are generic to all common epilepsy types, but additionally undergo syndrome‐specific modulation.

The neurobiological basis of these observations is at present unclear, but could relate to the underlying severity and extent of cortical dysfunction. Evidence from different lines of research gives strength to the idea that there are indeed generic disease mechanisms or processes that cut across epilepsy phenotypes, and that might affect oscillatory EEG features. For instance, it is well‐known from clinical experience that multiple pathologies lead to similar seizure behavior, a phenomenon which can be explained by the involvement of large‐scale networks.^11^ Indeed, a recent MRI study has shown that common epilepsy syndromes share structural abnormalities most prominently in right thalamus and precentral gyri,^28^ and an overview of recent neuroimaging studies suggest that medial frontal cortices are key nodes in epilepsy‐related networks as well.^29^ Interestingly, the topographies of alpha‐power shift we uncovered in PSC patients are broadly reminiscent of those associated with mid‐frontal theta oscillations (4–7 Hz) seen during cognitive tasks; these are commonly thought to arise from medial frontal cortices.^30^ Recent results from intracranial EEG suggest that the alpha rhythm is generated by anterior (“higher‐order”) cortices and then travels posteriorly in a wave‐like fashion, driving thalamic alpha‐rhythm as well.^1^ With this background in mind, one might therefore hypothesize that alpha‐power shifts might reflect dysfunction of a large‐scale cortico‐thalamic network that includes frontal cortex, and that disruption of this circuitry might be the common final pathway that links disparate clinical syndromes. This hypothesis could be tested with concurrent EEG‐fMRI data, or by correlating EEG data with MRI‐based cortex morphometry. Whether alpha‐power shifts are indeed causally involved in seizure liability could be further assessed in longitudinal studies: our results would predict that the alpha rhythm should shift back to normal frequency and configuration after treatment initiation, and that this effect might depend on the degree of underlying structural compromise.

Limitations of this study include its heterogeneous in‐ and outpatient sample, and its reliance on seizure self‐reports, which probably underestimate seizure occurrence. Barring invasive long‐term recordings,^31^ there is currently no other reliable approach to assess the ground truth of seizure frequency in any single patient. Scalp video‐telemetry, particularly at home, might offer a solution, but seizure rates in this context will likely depend on a number of circumstantial factors, for example timing and tempo of AED weaning, and recording length. On the other hand, we note that we found a direct correlation between seizure frequency and alpha‐power shifts (see Data S1). This indicates that, despite the shortcomings of seizure self‐reports, our approach was sensitive enough to capture meaningful (continuous) variability between individual seizure load and alpha‐rhythm abnormalities. Another concern is that our study had less power to detect interactions between syndrome type and levels of seizure control, given the low number of patients in each subgroup. However, we note effects for the different subgroup contrasts were large compared to the interaction contrast, which had an exceedingly low effect size (*η* ^*2*^ = 0.006). It seems therefore unlikely that we missed an interaction in the present data set. Finally, excessive daytime sleepiness is a well‐known phenomenon in patients with epilepsy,^32^ and sleep homeostasis might be particularly disrupted in drug‐resistant patients.^33^ Since we did not assess sleepiness explicitly, it cannot be definitively ruled out, and we think it should be recorded systematically in future studies (e.g., with patient questionnaires). Even so, given that our analyses ruled out circadian differences in EEG recordings, and the fact that experienced neurophysiologists selected the analyzed EEG segments, it seems highly unlikely that sleepiness played a major role in our data.

In sum, we show that the resting‐state human alpha rhythm is slower and extends frontally in patients with epilepsy, a phenomenon that we were able to link to seizure control across clinical syndrome boundaries. While the pathophysiology of such resting‐state alterations remains to be explored,^34^ we hypothesize that this effect could be commensurate with the degree of cortico‐thalamic dysfunction. This type of analysis could therefore provide a gateway to understand the pathophysiology of epilepsy or to develop epilepsy biomarkers that does not depend on the recording of epileptiform signals.

## Author Contributions

A.P. and E.A. conceived of the study. E. A. participated in collection of clinical data, wrote all software code, performed all data analyses, interpreted results and drafted/revised the manuscript, tables and figures. A.P. collected and curated all clinical and EEG data, drafted/reviewed the manuscript. C.T. and S.N.Y. participated in data analysis and reviewed the manuscript. F.B. and R.D.C.E helped with visual EEG data analysis and selection. M.P.R. supervised the study and its design, interpreted data, and revised manuscript and figures.

## Conflict of Interest

None declared.

## Supporting information


**Data S1.** Document describing rationale, methods and results of supplemental control analyses.
