# Supplementary material for: Slower alpha rhythm associates with poorer seizure control in epilepsy
Source: Ann Clin Transl Neurol. 2018 Dec 18;6(2):333–43. doi: 10.1002/acn3.710 (PMC6389754; doi:10.1002/acn3.710)
Supplement: Supplementary file 1 — Data S1. Document describing rationale, methods and results of supplemental control analyses. [file ACN3-6-333-s001.docx]

**Supplemental Materials**

**Content**

This document describes supplementary analyses to Abela, Pawley et al., “Slower alpha rhythm associates with poorer seizure control in epilepsy”. The order of analyses is as follows:

**Analysis 1** Re-analysis using individual alpha frequencies (IAF) instead of alpha-power shift.

**Analysis 2** Test of reproducibility using a second segment of EEG data.

**Analysis 3** Assessment of two alternative seizure-control definitions, i.e.

(a) Good seizure control: no seizures versus poor seizure control: any seizures,

(b) Good seizure control: below median of seizure frequency versus poor seizure control: above median of seizure frequency.

**Analysis 4** Correlation analysis using a continuous measure of seizure frequency

**Analysis 5** Comparing EEG recording times across groups to assess potential circadian effects

**Analysis 6** Additional control for anti-epileptic drug (AED) effects.

**Analysis 7** Topographic analysis to rule out lesional effects on spatial alpha-power shifts

All statistical analyses were done in JASP (Version 0.8.6, <https://jasp-stats.org/>), if not noted otherwise. Links point to extended results on the Open Science Framework ([www.osf.io)](http://www.osf.io)).

**Analysis 1: Re-analysis using individual alpha frequencies (IAF) instead of alpha-power shift.**

*Rationale and method*

We sought to confirm that alpha-power shifts were indeed accompanied by a deceleration of the individual alpha frequency (IAF). Because we calculated the alpha-power shift as a ratio between fixed frequency bands, a significant shift could occur due to redistribution of power between bands, without actual slowing of the peak individual alpha rhythm. We thus found the IAF as the frequency of maximum power in the extended alpha-band (6-13Hz), and compared individual log-transformed IAF between patient groups, using the same ANCOVA model described in the main manuscript. Our hypothesis was that, as in the main analysis, IAF would differ between seizure-control groups, but that there would not be an interaction between syndrome type (focal versus generalised) and seizure control.

*Results* (Link: <https://osf.io/739qr/>)

| **ANCOVA - IAF (log)** | | | | | | | | | | | | | | | | | | | | | | |
| --- | --- | --- | --- | --- | --- | --- | --- | --- | --- | --- | --- | --- | --- | --- | --- | --- | --- | --- | --- | --- | --- | --- |
| **Cases** | | | | | | **Sum of Squares** | | | **df** | | | **Mean Square** | | | | **F** | | | | **p** | | |
| Seizure control | | | | |  | 0.037 | |  | 1.000 | |  | 0.037 | | |  | 7.283 | | |  | 0.009 | |  |
| Syndrome | | | | |  | 0.016 | |  | 1.000 | |  | 0.016 | | |  | 3.052 | | |  | 0.086 | |  |
| Seizure control ✻ Syndrome | | | | |  | 2.352e -4 | |  | 1.000 | |  | 2.352e -4 | | |  | 0.046 | | |  | 0.831 | |  |
| Gender | | | | |  | 0.005 | |  | 1.000 | |  | 0.005 | | |  | 1.067 | | |  | 0.306 | |  |
| Age | | | | |  | 6.955e -5 | |  | 1.000 | |  | 6.955e -5 | | |  | 0.014 | | |  | 0.907 | |  |
| AED Load | | | | |  | 0.001 | |  | 1.000 | |  | 0.001 | | |  | 0.285 | | |  | 0.596 | |  |
| Residual | | | | |  | 0.286 | |  | 56.000 | |  | 0.005 | | |  |  | | |  |  | |  |
|  | | | | | | | | | | | | | | | | | | | | | | |
| *Note.*  Type III Sum of Squares | | | | | | | | | | | | | | | | | | | | | | |
| **Post Hoc Comparisons - Seizure control** | | | | | | | | | | | | | | | | | | | | | | |
|  | |  | | **Mean Difference** | | | | | | **SE** | | | | **t** | | | | **p _bonf_** | | | | |
| GSC |  | PSC |  | 0.053 | | |  | | | 0.020 | | |  | 2.699 | | |  | 0.009 | | |  | |
|  | | | | | | | | | | | | | | | | | | | | | | |

| **Post Hoc Comparisons - Syndrome** | | | | | | | | | | | |
| --- | --- | --- | --- | --- | --- | --- | --- | --- | --- | --- | --- |
|  | |  | | **Mean Difference** | | **SE** | | **t** | | **p _bonf_** | |
| FE |  | IGE |  | -0.035 |  | 0.020 |  | -1.747 |  | 0.086 |  |
|  | | | | | | | | | | | |

*Conclusion*

This analysis confirms that the alpha rhythm is indeed slower between seizure control groups. In contrast to the main analysis, there is no difference between FE and IGE, supporting the idea that slower alpha rhythm in epilepsy is syndrome-independent.

**Analysis 2: Test of reproducibility using a second segment of EEG data.**

*Rationale and method*

We assessed whether results differed between data segments. We thus repeated alpha-power shift comparisons for the average power spectra on a second data segment, which was available in 57/63 patients (missing in six because of considerable artefacts throughout the recording: 2 IGE, each in the GSC and PSC group, and 4 FE, 1 in the GSC and 3 in the PSC group). We applied the same ANCOVA model described in the main text.

*Results* (Link: <https://osf.io/739qr/>)

| **ANCOVA - Alpha-power shift (log) second segment** | | | | | | | | | | | | | | | | | | | | | | | | |
| --- | --- | --- | --- | --- | --- | --- | --- | --- | --- | --- | --- | --- | --- | --- | --- | --- | --- | --- | --- | --- | --- | --- | --- | --- |
| **Cases** | | | | | | **Sum of Squares** | | | | **df** | | | | **Mean Square** | | | | **F** | | | | **p** | | |
| Seizure control | | | | |  | 1.702 | | |  | 1.000 | | |  | 1.702 | | |  | 9.903 | | |  | 0.003 | |  |
| Syndrome | | | | |  | 0.947 | | |  | 1.000 | | |  | 0.947 | | |  | 5.508 | | |  | 0.023 | |  |
| Syndrome ✻ Seizure control | | | | |  | 0.277 | | |  | 1.000 | | |  | 0.277 | | |  | 1.609 | | |  | 0.210 | |  |
| Gender | | | | |  | 0.351 | | |  | 1.000 | | |  | 0.351 | | |  | 2.044 | | |  | 0.159 | |  |
| Age | | | | |  | 0.007 | | |  | 1.000 | | |  | 0.007 | | |  | 0.042 | | |  | 0.839 | |  |
| AED Load | | | | |  | 0.602 | | |  | 1.000 | | |  | 0.602 | | |  | 3.503 | | |  | 0.067 | |  |
| Residual | | | | |  | 8.596 | | |  | 50.000 | | |  | 0.172 | | |  |  | | |  |  | |  |
|  | | | | | | | | | | | | | | | | | | | | | | | | |
| Note.  Type III Sum of Squares   \| **Post Hoc Comparisons - Seizure control** \| \| \| \| \| \| \| \| \| \| \| \| \| \| \| --- \| --- \| --- \| --- \| --- \| --- \| --- \| --- \| --- \| --- \| --- \| --- \| --- \| --- \| \|  \| \|  \| \| **Mean Difference** \| \| **SE** \| \| **t** \| \| **Cohen's d** \| \| **p _bonf_** \| \| \| GSC \|  \| PSC \|  \| -0.381 \|  \| 0.121 \|  \| -3.147 \|  \| -0.852 \|  \| 0.003 \|  \| \|  \| \| \| \| \| \| \| \| \| \| \| \| \| \| \| *Note.*  Cohen's d does not correct for multiple comparisons. \| \| \| \| \| \| \| \| \| \| \| \| \| \| | | | | | | | | | | | | | | | | | | | | | | | | |
| **Post Hoc Comparisons - Syndrome** | | | | | | | | | | | | | | | | | | | | | | | | |
|  | |  | | **Mean Difference** | | | | **SE** | | | | **t** | | | | **Cohen's d** | | | | **p _bonf_** | | | | |
| FE |  | IGE |  | 0.287 | | |  | 0.122 | | |  | 2.347 | | |  | 0.633 | | |  | 0.023 | | |  | |
|  | | | | | | | | | | | | | | | | | | | | | | | | |
| *Note.*  Cohen's d does not correct for multiple comparisons. | | | | | | | | | | | | | | | | | | | | | | | | |

*Conclusion*

This analysis confirms that alpha-power is shifted towards lower frequencies in a second data set from the same recording, and thus reproducible within one recording session.

**Analysis 3: Assessment of two alternative seizure-control definitions**

*Rationale and method*

Because we used a heuristic for dividing patients into seizure-control subgroups, there is a concern that group differences might be sensitive to the seizure frequency thresholds used. Also, patients that are completely seizure free might differ neurobiologically from those with only a few seizures. We therefore tested two alternative seizure control definitions: a zero-split (i.e. patients without any seizures versus all others) and a median-split (i.e. patients with seizure frequency below versus above the median). We applied the same ANCOVA model described in the main text.

*Results* (Link: <https://osf.io/ch78y/>)

| **ANCOVA - Alpha-power shift (log) / Zero-split** | | | | | | | | | | | | | | | | | | | | | | | | |
| --- | --- | --- | --- | --- | --- | --- | --- | --- | --- | --- | --- | --- | --- | --- | --- | --- | --- | --- | --- | --- | --- | --- | --- | --- |
| **Cases** | | | | | | **Sum of Squares** | | | | **df** | | **Mean Square** | | | | **F** | | | | **p** | | **η²** | | |
| Seizure control (zero split) | | | | |  | 1.471 | | |  | 1.000 |  | 1.471 | | |  | 7.441 | | |  | 0.009 |  | 0.103 | |  |
| Syndrome | | | | |  | 1.333 | | |  | 1.000 |  | 1.333 | | |  | 6.741 | | |  | 0.012 |  | 0.093 | |  |
| Seizure control (zero split) ✻ Syndrome | | | | |  | 0.121 | | |  | 1.000 |  | 0.121 | | |  | 0.610 | | |  | 0.438 |  | 0.008 | |  |
| Gender | | | | |  | 0.015 | | |  | 1.000 |  | 0.015 | | |  | 0.074 | | |  | 0.787 |  | 0.001 | |  |
| Age | | | | |  | 0.008 | | |  | 1.000 |  | 0.008 | | |  | 0.040 | | |  | 0.842 |  | 0.001 | |  |
| AED Load | | | | |  | 0.258 | | |  | 1.000 |  | 0.258 | | |  | 1.307 | | |  | 0.258 |  | 0.018 | |  |
| Residual | | | | |  | 11.072 | | |  | 56.000 |  | 0.198 | | |  |  | | |  |  |  |  | |  |
|  | | | | | | | | | | | | | | | | | | | | | | | | |
| Note.  Type III Sum of Squares | | | | | | | | | | | | | | | | | | | | | | | | |
| **Post Hoc Comparisons - Seizure control (Zero split)** | | | | | | | | | | | | | | | | | | | | | | | | |
|  | |  | | **Mean Difference** | | | | **SE** | | | | | | **t** | | | | **p _bonf_** | | | | | | |
| GSC |  | PSC |  | -0.339 | | |  | 0.124 | | | | |  | -2.728 | | |  | 0.009 | | | | |  | |
|  | | | | | | | | | | | | | | | | | | | | | | | | |

| **Post Hoc Comparisons - Syndrome** | | | | | | | | | | | |
| --- | --- | --- | --- | --- | --- | --- | --- | --- | --- | --- | --- |
|  | |  | | **Mean Difference** | | **SE** | | **t** | | **p _bonf_** | |
| FE |  | IGE |  | 0.325 |  | 0.125 |  | 2.596 |  | 0.012 |  |
|  | | | | | | | | | | | |

(continued on next page)

| **ANCOVA - Alpha-power shift (log) / Median-split** | | | | | | | | | | | | | |
| --- | --- | --- | --- | --- | --- | --- | --- | --- | --- | --- | --- | --- | --- |
| **Cases** | | **Sum of Squares** | | **df** | | **Mean Square** | | **F** | | **p** | | **η²** | |
| Seizure control (median split) |  | 1.742 |  | 1.000 |  | 1.742 |  | 8.940 |  | 0.004 |  | 0.121 |  |
| Syndrome |  | 1.450 |  | 1.000 |  | 1.450 |  | 7.438 |  | 0.009 |  | 0.101 |  |
| Seizure control (median split) ✻ Syndrome |  | 5.230e -4 |  | 1.000 |  | 5.230e -4 |  | 0.003 |  | 0.959 |  | 0.000 |  |
| Gender |  | 0.016 |  | 1.000 |  | 0.016 |  | 0.081 |  | 0.777 |  | 0.001 |  |
| Age |  | 0.037 |  | 1.000 |  | 0.037 |  | 0.189 |  | 0.665 |  | 0.003 |  |
| AED Load |  | 0.195 |  | 1.000 |  | 0.195 |  | 1.001 |  | 0.321 |  | 0.014 |  |
| Residual |  | 10.913 |  | 56.000 |  | 0.195 |  |  |  |  |  |  |  |
|  | | | | | | | | | | | | | |
| Note.  Type III Sum of Squares | | | | | | | | | | | | | |

| **Post Hoc Comparisons - Seizure control (Median split)** | | | | | | | | | | | |
| --- | --- | --- | --- | --- | --- | --- | --- | --- | --- | --- | --- |
|  | |  | | **Mean Difference** | | **SE** | | **t** | | **p _bonf_** | |
| GSC |  | PSC |  | -0.356 |  | 0.119 |  | -2.990 |  | 0.004 |  |
|  | | | | | | | | | | | |

| **Post Hoc Comparisons - Syndrome** | | | | | | | | | | | |
| --- | --- | --- | --- | --- | --- | --- | --- | --- | --- | --- | --- |
|  | |  | | **Mean Difference** | | **SE** | | **t** | | **p _bonf_** | |
| FE |  | IGE |  | 0.333 |  | 0.122 |  | 2.727 |  | 0.009 |  |
|  | | | | | | | | | | | |

*Conclusion*

Two alternative seizure-control definitions yield qualitatively similar results to the main analysis, indicating that findings are not sensitive to the choice of threshold.

**Analysis 4: Correlation analysis using a continuous measure of seizure frequency**

*Rationale and method*

All analyses above and in the main manuscript were based on dichotomisation of patient groups based on seizure frequency. While results are consistent across three different criteria, they do not reveal the full extent of the variability in the data. To gain further insight into the relationship between alpha-rhythm alterations and seizure control, we calculated Pearson’s correlation between individual alpha-power shift and self-reported seizure frequency across the patient cohort, without dichotomisation, both using channel-averaged and topographically-resolved alpha-power shift values. Both measures were log-transformed before correlation. An offset of 1 was added to the seizure frequency values before taking the log to avoid zero values. The topographical analysis with the same permutation-based methods are described in the main manuscript.

*Results* (Link: <https://osf.io/npdx9/>)

Average alpha-power shift across all channels was positively correlated with individual seizure frequency: Person’s correlation coefficient, r = 0.403, p <.001 (Figure, left panel). Using spatial correlations (seizure frequency against alpha-power shift at each channel), we found a positive correlation, particularly over frontal recording sites (Figure, right panel).


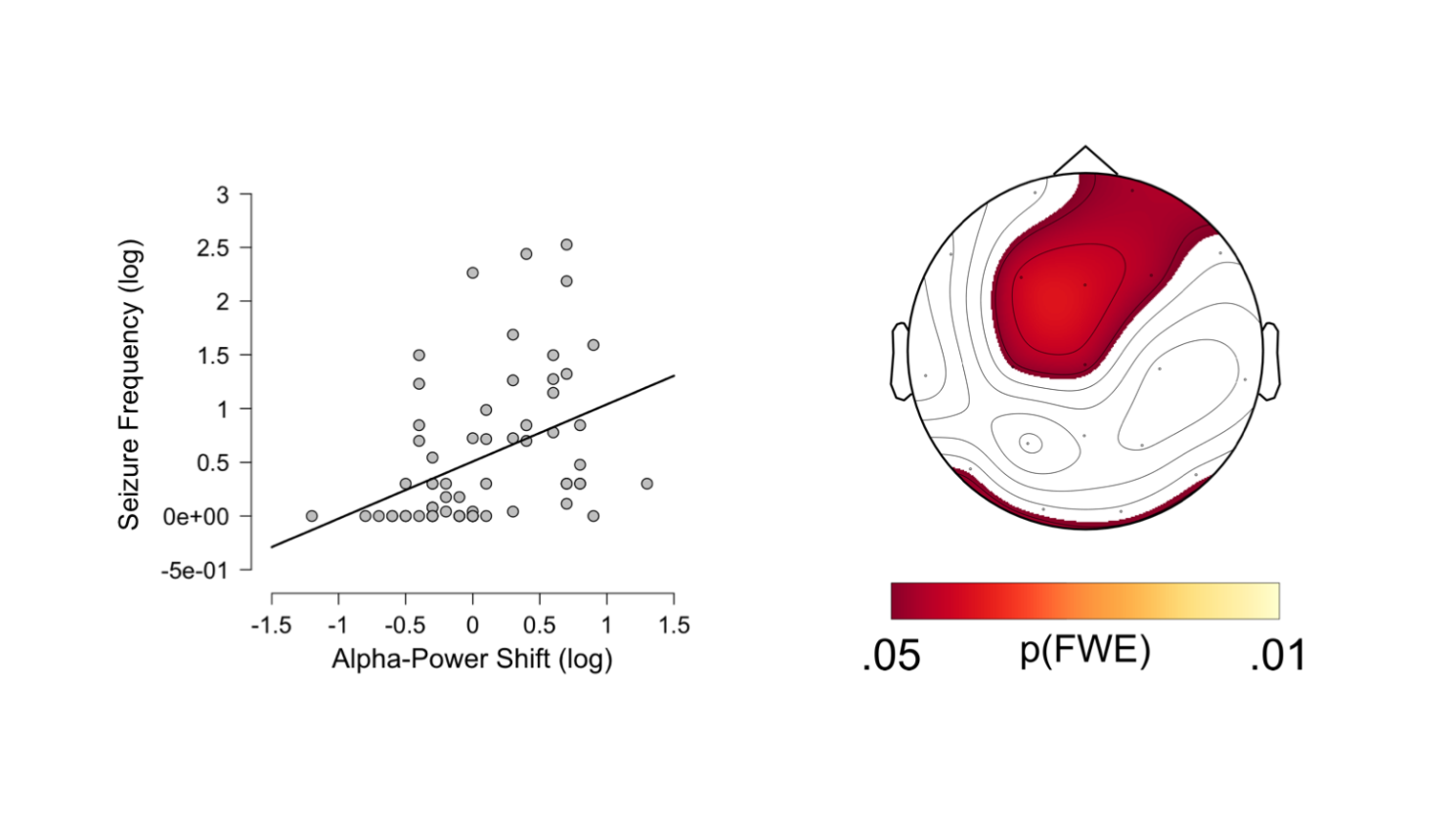


*Conclusion*

Correlation analysis yields qualitatively similar results to the main subgroup analyses: poorer seizure control associates with an increased shift of alpha-power to lower frequencies, particularly over frontal recordings sites.

**Analysis 5: Comparing EEG recording times across groups to assess potential circadian effects.**

*Rationale and method*

We investigated whether there were systematic differences between subgroups in the time of day at which EEG recording was carried out. This was done because it was not possible to record all EEG data during the same time of the day, and because there is well-documented interplay between circadian rhythms and seizure liability^1,2^. To assess this, we retrieved the time stamps of each EEG recording from the raw data files, converted them from hours to radians (on a 24-hr circle) and calculated a Watson-Williams test, which compares means of circular data for two or more groups, between GSC and PSC patients (CircStat, <https://github.com/circstat/circstat-matlab>)^3^.

*Results*

**Descriptive Statistics**

| Group | Circular Mean (rad.) | Circular Standard Deviation (rad.) |
| --- | --- | --- |
| GSC | -2.7002 | 0.6427 |
| PSC | -2.5939 | 0.7523 |

**ANOVA-Table (Watson-Williams test)**

|  |  | d. f. | SS |  | MS | F | p-Value |
| --- | --- | --- | --- | --- | --- | --- | --- |
| Columns |  | 1 | 0.07 | 0.07 | 0.30 | 0.5861 | 0.5861 |
| Residual |  | 61 | 15.69 | 0.26 |  |  |  |
| Total |  | 62 | 15.75 |  |  |  |  |

d. f, degrees of freedom; SS, sum of squares; MS, mean square; F, F-statistic

*Conclusion*

Analysis of recording times using circular statistics shows no difference in daytime of EEG recordings between seizure control groups, thus rendering circadian effects unlikely.

**Analysis 6: Additional control for anti-epileptic drug (AED) effects.**

*Rationale and method*

We found that patients with good seizure control (GSC) were treated on average with higher doses of lamotrigine (LTG), which has been shown to accelerate alpha frequency, and might therefore *reduce* the alpha-power shift measure^4^. To test whether different LTG dosages biased between-group differences, we calculated independent samples t-test between (1) all GSC patients (n = 10) and poor seizure control (PSC) patients (n = 12) on LTG, (2) GSC patients with (n=10) and without (n=15) LTG, and (3) PSC patients with (n = 12) and without (n = 26) LTG. We hypothesised that, if the first comparison yielded a difference, but the other two did not, then the difference between groups would likely be driven by seizure control, and not by an LTG acceleration effect. Put differently, if LTG were to accelerate alpha rhythms in a measurable way in our cohort, one would expect both GSC and PSC patients on LTG to have a lower alpha-power shift (i.e. more high alpha-power) than their non-LTG counterparts. For completeness, we also compared alpha-power shifts in all patients without LTG (Test 4).

*Results* (Link: <https://osf.io/ruvf6/>)

**Test 1: all GSC patients on LTG versus all PSC patients on LTG**

| **Group Descriptives** | | | | | | | | | | | |
| --- | --- | --- | --- | --- | --- | --- | --- | --- | --- | --- | --- |
|  | | **Group** | | **N** | | **Mean** | | **SD** | | **SE** | |
| AlphaPowerShift(log) |  | GSC |  | 10 |  | -0.160 |  | 0.212 |  | 0.067 |  |
|  |  | PSC |  | 10 |  | 0.420 |  | 0.577 |  | 0.182 |  |
|  | | | | | | | | | | | |

| **Independent Samples T-Test: all GSC patients on LTG versus all PSC patients on LTG** | | | | | | | | | |
| --- | --- | --- | --- | --- | --- | --- | --- | --- | --- |
|  | | **Test** | | **Statistic** | | **df** | | **p** | |
| AlphaPowerShift(log) |  | Student |  | -2.984 |  | 18.000 |  | 0.008 |  |
|  |  | Welch |  | -2.984 |  | 11.384 |  | 0.012 |  |
|  | | | | | | | | | |

**Test 2: GSC patients on LTG versus GSC patients without LTG**

| **Group Descriptives** | | | | | | | | | | | |
| --- | --- | --- | --- | --- | --- | --- | --- | --- | --- | --- | --- |
|  | | **Group** | | **N** | | **Mean** | | **SD** | | **SE** | |
| AlphaPowerShift(log) |  | No |  | 15 |  | -0.300 |  | 0.478 |  | 0.123 |  |
|  |  | Yes |  | 10 |  | -0.160 |  | 0.212 |  | 0.067 |  |
|  | | | | | | | | | | | |

| **Independent Samples T-Test** | | | | | | | |
| --- | --- | --- | --- | --- | --- | --- | --- |
|  | | **t** | | **df** | | **p** | |
| AlphaPowerShift(log) |  | -0.866 |  | 23.000 |  | 0.395 |  |
|  | | | | | | | |
| Note.  Student's t-test. | | | | | | | |

(continued on next page)

**Test 3: PSC patients on LTG versus PSC patients without LTG**

| **Group Descriptives** | | | | | | | | | | | |
| --- | --- | --- | --- | --- | --- | --- | --- | --- | --- | --- | --- |
|  | | **Group** | | **N** | | **Mean** | | **SD** | | **SE** | |
| AlphaPowerShift(log) |  | No |  | 26 |  | 0.231 |  | 0.432 |  | 0.085 |  |
|  |  | Yes |  | 12 |  | 0.417 |  | 0.564 |  | 0.163 |  |
|  | | | | | | | | | | | |

| **Independent Samples T-Test** | | | | | | | |
| --- | --- | --- | --- | --- | --- | --- | --- |
|  | | **t** | | **df** | | **p** | |
| AlphaPowerShift(log) |  | -1.118 |  | 36.000 |  | 0.271 |  |
|  | | | | | | | |
| Note.  Student's t-test. | | | | | | | |

**Test 4:** **all GSC patients not on LTG versus all PSC patients not on LTG**

| **Group Descriptives** | | | | | | | | | | | |
| --- | --- | --- | --- | --- | --- | --- | --- | --- | --- | --- | --- |
|  | | **Group** | | **N** | | **Mean** | | **SD** | | **SE** | |
| AlphaPowerShift(log) |  | GSC |  | 15 |  | -0.300 |  | 0.478 |  | 0.123 |  |
|  |  | PSC |  | 27 |  | 0.222 |  | 0.434 |  | 0.084 |  |
|  | | | | | | | | | | | |

| **Independent Samples T-Test** | | | | | | | |
| --- | --- | --- | --- | --- | --- | --- | --- |
|  | | **t** | | **df** | | **p** | |
| AlphaPowerShift(log) |  | -3.602 |  | 40.00 |  | < .001 |  |
|  | | | | | | | |
| Note.  Student's t-test. | | | | | | | |

*Conclusion*

This set of analyses shows that, while well-controlled patients on LTG had indeed a lower alpha-power shift compared to poorly controlled patients on LTG (Test 1), they did not differ on average from their peers without LTG (Test 2). This was replicated in the poorly controlled patients as well (Test 3), indicating that LTG did not bias our main results; i.e. LTG did not artificially reduce the alpha-power shift and therefore enhance differences between seizure control groups. This also confirmed with the last test (Test 4): patients off-LTG show the same pattern compared to those on-LTG (Test 1).

We note that small subject numbers are an important limitation, and we cannot rule out significant LTG effects on alpha power in larger or more homogeneous populations (i.e. patients in monotherapy).

**Analysis 7: Topographic analysis to rule out lesional effects on spatial alpha-power shifts**

*Rationale and method*

We show an anteriorisation of the slow alpha rhythm that is particularly pronounced in patients with focal epilepsies (see Figure 2 in the main manuscript). Because this group naturally presents a higher rate of lesional syndromes, the anteriorisation could be an epiphenomenon. To rule out this possibility, we calculated spatial permutation-based statistics (cf. Methods in main manuscript) in two subgroups without lesions: poorly controlled idiopathic generalised epilepsy (IGE, n = 10) and poorly controlled non-lesional focal epilepsy (FE, n=11) patients, both against healthy controls.

*Results*

The figure below shows scalp topographic maps of family-wise error (FWE)-corrected p-values for two independent-sample t-tests of alpha-power shift: IGE patients with poor seizure control against healthy controls (left), and non-lesional FE patients with poor seizure control against healthy controls (right). In both cases, significant effects can be seen in frontal regions, although the effect is considerably stronger and has a much wider spatial distribution in FE patients.


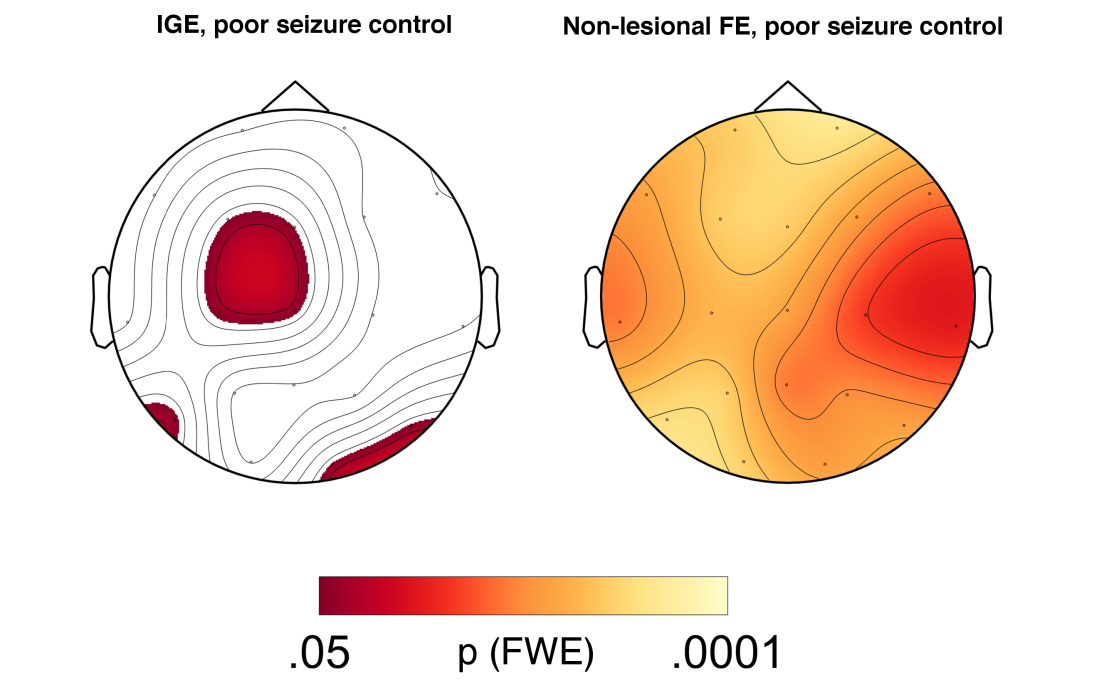


*Conclusion*

This analysis shows that anteriorisation of low-frequency alpha-power in poorly controlled patients does not depend on the presence of lesions, since both IGE patients (which are per definition non-lesional) and FE patients without discernible lesions on neuroimaging show this effect. However, the magnitude of the effect is conspicuously different, either due to low statistical power or differing pathophysiological mechanisms, or both.

**References**

1. Baud MO, Kleen JK, Mirro EA, et al. Multi-day rhythms modulate seizure risk in epilepsy. Nat. Commun. 2018;9(1):88.

2. Quigg M. Circadian rhythms: interactions with seizures and epilepsy. Epilepsy Res. 2000;42(1):43–55.

3. Berens P, Baclawski K, Berens P, Baclawski K. CircStat: A MATLAB Toolbox for Circular Statistics. J. Stat. Softw. 2009;30(April):1–3.

4. Clemens B, Ménes A, Piros P, et al. Quantitative EEG effects of carbamazepine, oxcarbazepine, valproate, lamotrigine, and possible clinical relevance of the findings. Epilepsy Res. 2006;70(2–3):190–199.
